# Establishing the Minimum Media Time Sample Required to Obtain Reliable Estimates of Children’s Digital Media Food Marketing Exposures

**DOI:** 10.1016/j.cdnut.2023.100092

**Published:** 2023-04-25

**Authors:** Emily Nicholson, Bridget Kelly

**Affiliations:** Early Start, School of Health and Society, Faculty of the Arts, Social Sciences and Humanities, University of Wollongong, Wollongong, New South Wales, Australia

**Keywords:** food, marketing, digital, online, monitoring

## Abstract

**Background:**

The ubiquitous nature of food marketing on digital media likely has a profound effect on children’s food preferences and intake. Monitoring children’s exposure to digital marketing is necessary to raise awareness of the issue, inform policy development, and evaluate policy implementation and effect.

**Objectives:**

This study aimed to establish whether smaller time samples (less time and/or fewer days captured) would provide robust estimates of children’s usual exposures to food marketing.

**Methods:**

Using an existing data set of children’s digital marketing exposures, which captured children’s total screen use over 3 d, a reliability assessment was performed.

**Results:**

A subsample of 30% of children’s usual screen time was found to provide reliable estimates of digital food marketing exposure compared with the full sample (intraclass correlation coefficient: 0.885; Cronbach α: 0.884). There was no difference in the rates of marketing (exposures/h) between weekdays and weekend days.

**Conclusions:**

These findings enable researchers to reduce the time and resource constraints that have previously restricted this type of monitoring research. The reduced media time sample will further lessen participant burden.

## Introduction

It is widely acknowledged that children’s (defined as younger than 18 y) exposure to food marketing significantly influences their food preferences, purchase requests, and food intake [1,2]. Most of the promoted foods and beverages are considered unhealthy or high in fat, added sugar and/or salt [2]. This has led to global mandates for policy action to protect children from the harmful effects of unhealthy food marketing from international health agencies [3,4]. At the time of writing, the WHO had released draft guidelines that recommended member states to implement mandatory policies to restrict food marketing to which children are exposed [5]. The growth of digital media use in the past decade has seen a shift in marketing techniques, with increased advertising expenditure being directed into digital marketing [6,7]. Digital media has enabled marketers to reach children in a more personalized and authentic way, whereas infiltrating their messages across numerous platforms simultaneously [8]. This has created challenges for children in recognizing this marketing [9] and for researchers in monitoring children’s exposure to it [10,11]. Young people’s exposure to, and engagement with, food marketing in online media has been associated with heighted effects on food choice and consumption behaviors when compared with other media exposures (namely, on television) [12,13].

Monitoring evidence is necessary to raise awareness around the extent of a problem, frame policy discourse, evaluate compliance and effectiveness of policies, and hold governments and industries to consider marketing practices [14]. Monitoring frameworks have been developed internationally to support researchers in undertaking studies to measure the exposure and power of food marketing to children, notably INFORMAS and the WHO CLICK framework [15,16]. The WHO CLICK monitoring framework is a 5-step process designed to gather data on children’s exposure to digital marketing. INFORMAS protocols for monitoring food marketing on television [17] and in outdoor settings [18] have been adopted in >10 studies in the past 6 y [[19], [20], [21], [22], [23], [24], [25], [26], [27], [28], [29], [30], [31], [32]]. This has enabled consistency in study methods, promoting the creation of comparable data across countries and over time. Approaches to monitor children’s digital food marketing exposure have spanned methods that assess children’s potential exposures, using content analyses of platforms that are popular among children [[33], [34], [35], [36]]; assess children’s estimated exposures, by combining content analyses with information on children’s use of digital platforms [[37], [38], [39]]; or assess children’s actual exposures, by capturing real-time data of individual children’s interactions with online platforms [[40], [41], [42], [43]]. Most studies have applied content analyses of webpages or online platforms to derive estimates of potential exposures, with relatively few studies considering the reach of, or children’s engagement with, digital marketing use on these platforms [15].

Studies assessing children’s actual food marketing exposures consider the individualized nature of behavioral marketing and require the collection of media data from individual children. These studies have previously required between 10 min and 1 wk of data collection and produce data in the form of photographs (or screenshots) and/or videos (or screen recordings) [[40], [41], [42], [43]]. This produces a large amount of data for researchers to code and analyze. One of the components of the WHO CLICK framework relates to data collection using real-time screen capture software on a sample of the study population to assess what children see online on their devices, collected over a few days [15]. This approach was based on an Australian study [40]. No further studies have yet been published based on this CLICK framework component, likely because it is resource intensive. Although these studies provide novel information on children’s actual exposure to, and engagement with, digital food marketing, they are time consuming to conduct, with high participant burden, and are, thus, less reproducible.

To address these practical limitations in applying screen capture methodologies to assess children’s actual exposures to food marketing online, this study aimed to explore the comparability of different media time samples, to identify whether smaller media samples can produce reliable estimates of children’s “usual” food marketing exposures on digital media. In addition, the study sought to determine whether it is necessary to capture weekdays and weekend days separately in estimating usual weekly exposure to digital food marketing. Findings from this reliability assessment can be used to determine an appropriate sampling approach for monitoring food marketing on digital media to provide robust estimates while minimizing respondent and researcher burden. Monitoring protocols that use reduced media time samples to assess children’s exposures to online food marketing will facilitate their adoption by researchers, including from less well-resourced countries, and support awareness raising and policy dialogs to protect children from this marketing.

## Methods

### Data source

A cross-sectional observational study conducted by Kelly et al. [40] in 2018–2019 aimed to quantify and describe Australian children’s exposure to digital marketing of food and drinks. The children, aged 13–17 y, recorded their mobile device screen for 2 weekdays and 1 weekend day any time they visited relevant web-based platforms and uploaded these recordings to a secure server at the conclusion of each day. Children were required to manually turn on the screen recording function on their device (using an application or setting, depending on the operating system) each time they went online. Participants uploaded between 1 and 67 video recordings each day. These recordings were watched by researchers at least twice to extract information on food marketing. The final sample of 95 children uploaded 267.8 h of video data, thus requiring >500 h of researcher time in coding. A prequestionnaire, completed by the participants before the screen capture component, captured data on participant’s self-reported usual time spent online on mobile devices for each day of the week (h or min). This was used to calculate their usual average weekday and weekend day online time on mobile devices. To be included in the study, participants had to record ≥30% of their usual time spent online (as reported in the prequestionnaire), on ≥1 weekday and 1 weekend day. The study was approved by the University of Wollongong Human Research Ethics Committee (HREC 2018/158). Written consent to participate was obtained from children and their parents.

### Procedure

The approach used was based on reliability assessments used in other fields of research, which aimed to determine the smallest possible sample that provides a reliable estimate when compared with a larger sample. This includes studies measuring usual physical activity behaviors, which have attempted to derive the minimum amount of time participants needed to wear accelerometers or pedometers to obtain reliable estimates of PALs [[44], [45], [46]].

**FIGURE 1:**
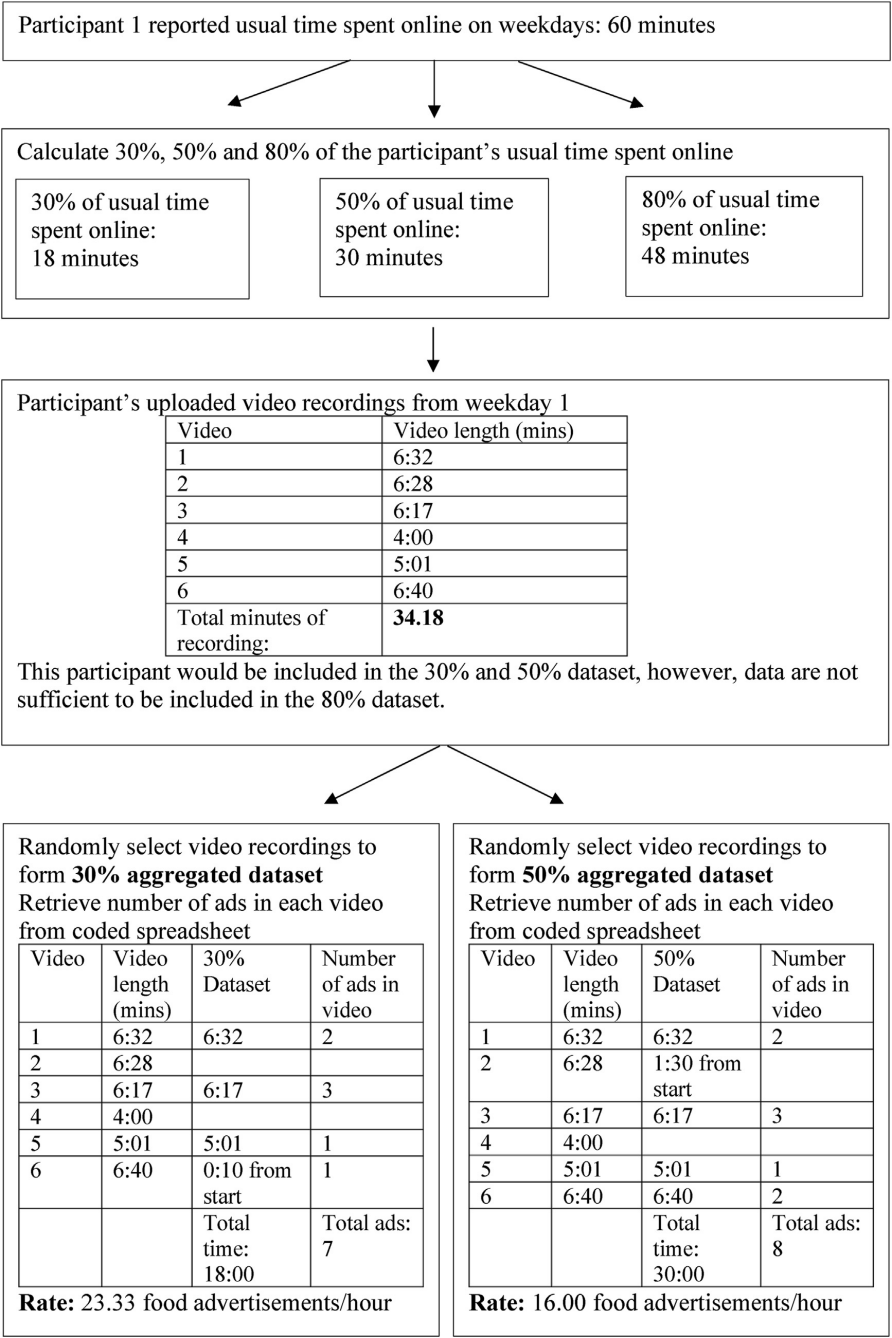
Example of the data manipulation procedure used to derive the different time sample data sets

Data for 92 participants were included in the reliability assessment. Each day of participant recording was treated as an individual case. Because some participants did not contribute 3 d of valid data (<30% of usual time online recorded), the total number of cases in the sample was 265 (Table 1). That is, each participant contributed between 1 and 3 d of data. Data from the prequestionnaire was used to calculate 30%, 50%, and 80% of a participant’s usual time spent online. This was used to create 3 new data sets by including video recordings that equated to 30%, 50%, and 80% of a child’s usual time spent online. The included video recordings were selected randomly from all videos for a child, using the randomize function in Microsoft Excel (Excel Version 16, 2021). The rate of marketing per hour was calculated for each individual case (day of recording) for the 30%, 50%, and 80% time sample data sets. An example of this procedure is shown in Figure 1.

**TABLE 1:** Grouped distribution of proportion of usual time spent online

Data set (% of usual time recorded)	Valid cases (*n*)	Median rate of marketing per hour (IQR)
30% of usual time	265	13.3 (5–27)
50% of usual time	179	14.0 (6–28)
80% of usual time	50	13.1 (3–23)

Additional analyses were conducted to determine whether there was a significant difference between the rate of food marketing per hour on weekdays and weekend days. This analysis was performed using the 30% aggregated data set because it had more valid cases than the 50% and 80% data sets and was deemed to appropriately estimate usual marketing exposures (based on the primary analyses). If a participant had 2 recordings for weekdays, the average rate of marketing across these days was calculated. Then, the average rate of marketing on weekdays was compared with the rate of marketing on weekend days to assess whether there was a statistically significant difference in marketing rates. All data preparation and manipulation were managed in Microsoft Excel.

### Statistical analysis

All statistical analyses were completed on IBM SPSS Statistics (version 28.0; IBM Corporation, Armonk, New York). Statistical significance was set at *P* < 0.05. Descriptive statistics for the rate of food marketing in each of the 3 data sets were computed. The rates of marketing did not meet normality assumptions based on visual inspection and the Shapiro-Wilk test of normality, and thus medians and interquartile ranges (IQRs) were reported. Reliability between data sets was measured using the intraclass correlation coefficient (ICC) and Cronbach α. Two cases were excluded from the reliability analyses because they had rates of marketing that were extreme outliers compared with the median rate of marketing in the sample. Reliability coefficients were tested between the 3 aggregated data sets (30%, 50%, and 80% of usual time spent online) and repeated to compare between only the 50% and 80% data sets. Bland–Altman plots were created to assess the mean differences and limits of agreement in the rate of marketing between 2 proportions of children’s usual time spent online. In this case, the smaller aggregated data sets (30% and 50% of usual time online) were compared separately with the larger data set (80% usual time online). The additional analyses used the Wilcoxon signed rank test to determine whether the rate of food marketing seen by the individual participants differed significantly on weekdays compared with that on weekend days.

## Results

### Descriptive statistics

With each reduced time sample data set, the rate of food marketing per hour of online recording remained relatively stable (Table 1). From the initial sample of 265 cases (days of recording), 179 represented ≥50% of usual time online, whereas 50 represented 80% of usual time online and were included in the respective data sets.

Despite the reduced number of cases in the 50% and 80% data sets, the demographic characteristics of participants in each data set were deemed to remain relatively stable and representative of the original sample (Supplemental Table 1). With the increased time of recording, the proportions of high socioeconomic status, male sex, and older participants (17–18 y) increased slightly. In our earlier analyses of children’s online food marketing exposure data [40], using negative binomial regression children’s age was not associated with their weekly exposure to food and beverage promotions. We re-ran this regression and confirmed that socioeconomic status and sex of participants were also not significantly associated with marketing exposures (*P* > 0.05). Participants who usually spent less time on their mobile devices were able to record a larger proportion of their usual time.

### Reliability between time sample data sets

Cronbach α and ICC across all 3 data sets revealed high levels of consistency (Table 2). The rate of marketing that participants were exposed to remained relatively stable from when 80% of their usual time online was included compared with that of when only 30% of their usual time online was included. The reliability coefficients improved when only the data sets representing 50% and 80% of usual time online were compared.

**TABLE 2:** Reliability between data sets representing different proportions of usual time spent online

Data sets used	Cases (*n*)	Cronbach α	Intraclass correlation coefficient (95% CI)
30%, 50%, and 80% of usual time data sets	48	0.884	0.885 (0.814, 0.931)
50% and 80% of usual time data sets	48	0.954	0.954 (0.918, 0.974)

**FIGURE 2:**
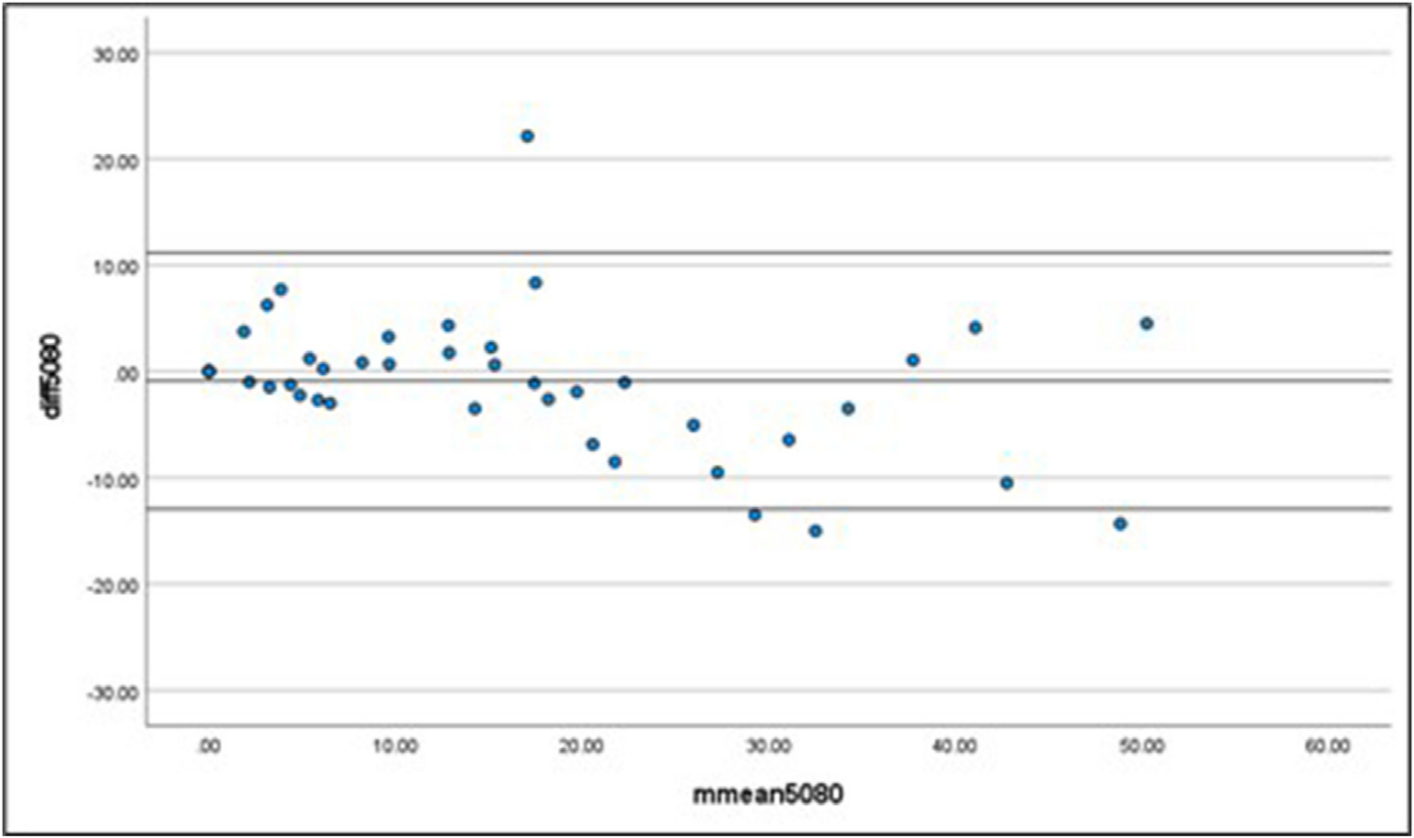
Bland–Altman plot representing a comparison between the 50% and 80% data sets.

**FIGURE 3:**
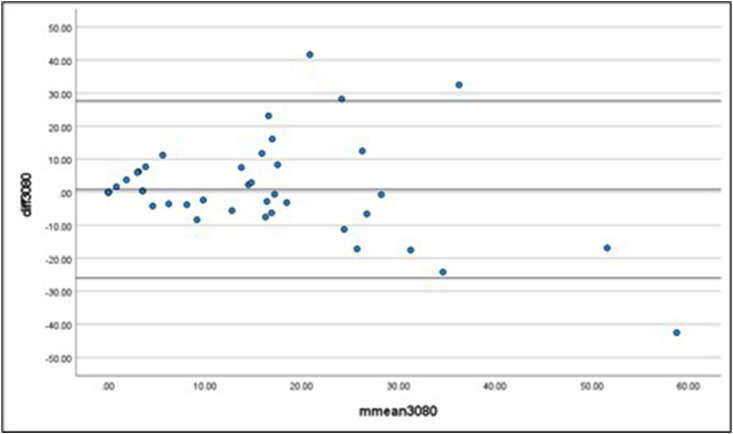
Bland–Altman plot representing a comparison between the 30% and 80% data sets.

The Bland–Altman plots demonstrated a good level of agreement between the data sets. The mean difference between the 80% data set and the 30% and 50% data sets were 0.83 advertisements/h (13.66) and −0.88 advertisements/h (6.15), respectively. The limits of agreement were smaller for the 50% compared with 80% data set (upper limit: 11.17; lower limit: −12.93). The larger upper and lower limits of agreement for the 30% compared with 80% data set (upper limit: 27.61; lower limit: −25.95) indicate the higher standard deviation of the mean difference. Although the 50% compared with 80% plot (Figure 2) illustrated a stronger overall agreement, the 30% compared with 80% plot (Figure 3) showed acceptable agreement between the 2 proportions of usual time spent online.

### Correlation between weekdays and weekend days

The median rate of food marketing per hour for weekdays was 15.0 (IQR: 6–29) compared with 11.1 (IQR: 3–27) for weekend days (*Z* = −1.518; *P* = 0.129).

## Discussion

This study sought to improve the feasibility of time sampling protocols for monitoring children’s actual exposure to food marketing on digital media. The reliability assessment identified a minimum sampling duration of 30% of children’s usual time online should be captured for robust estimates of usual digital food marketing exposures. This was evident from the acceptable reliability coefficients and agreement between data sets. This study also found no significant difference between the rates of food marketing per hour on weekdays and weekend days, suggesting that monitoring studies need to only capture either a weekday or weekend day. However, children’s media use does vary across days of the week [[47], [48], [49]], and therefore, effort should be taken to gather detailed information on children’s usual media use for the correct extrapolation of rates of marketing to derive usual marketing exposures (for example, per week). Measures of media use include surveys/questionnaires [[50], [51], [52]], media use diaries [53], and time use diaries [54,55].

The recent emergence of digital media marketing means this is a relatively understudied area. Monitoring techniques that have been used to assess children’s exposure to food marketing on broadcast media (namely television) are inappropriate for monitoring digital media because of to the personalized and targeted nature of marketing on digital platforms [7,10]. Gathering robust estimates of children’s actual exposure to food marketing on digital platforms requires real-time data collection of children’s interactions with these media. The results of this reliability assessment support the development of feasible protocols for monitoring children’s actual exposures to digital media by substantially reducing the time participants need to record their media use to 30% of usual time online for either 1 weekday or 1 weekend day. This is a departure from other suggested protocols [15,40], which will support uptake of monitoring in other jurisdictions and over time. An INFORMAS protocol for monitoring digital media marketing is currently being developed. The findings from this study will be valuable in supporting the sampling approach recommended in the protocol.

This research had a number of limitations. First, the aggregated data sets contained unequal sample sizes where less data were available for increased time of recording. This was because most participants recorded only a subsample of their usual time spent online. Hence, comparisons with the larger sampling duration data sets (that is, 80% data set) had notably less data for analysis, reducing the statistical power of the results. In addition, the 30%, 50%, and 80% data set increments were based on judgments around the distribution of the data, rather than in accordance with any previous research. The estimates of children’s usual time spent online were based on children’s self-report, which has been found to overestimate media use compared with other methods such as media diaries [51,53]. The mechanism that was used by participants to record their device screen required them to manually enable recording each time they went online. Although multiple reminder messages were sent to participants each day of recording, most participants recorded only a portion of their online time. Finally, the use of the survey panel to recruit participants may have led to selection biases. For example, half of the sample (51%) lived in areas of high social advantage.

In conclusion, the analyses suggest that monitoring studies that capture 1 d of data for 30% of children’s usual time spent online are adequate to assess children’s actual exposures to food marketing on digital platforms. This reduced sample will enable researchers to overcome financial, time, and resource constraints that restrict this type of research. The sampling approach also reduces participant burden, thereby reducing study attrition. These findings will provide researchers with a minimum sampling protocol to inform methodologies in future studies. Given the significant effects of children’s food marketing exposure on their diet-related outcomes and the global mandate for policies to restrict this marketing, the development of feasible approaches for monitoring children’s food marketing exposures are required to propel national governments’ policy actions and to assess compliance with such actions.

## Author contributions

The authors’ responsibilities were as follows—EN: planned and conducted the analyses, and drafted and revised the manuscript; BK: designed the research, led the collection of the data, supervised the research student in undertaking the analyses, reviewed the manuscript, and revised the manuscript after peer review; and both authors: read and approved the final manuscript.

## Data availability

The data described in the manuscript, code book, and analytic code will be made publicly and freely available without restriction at https://cloudstor.aarnet.edu.au/plus/s/7f1vf6Hw8vuh8Eh.

## Funding

The authors reported no funding received for this study.

## Author disclosures

EN and BK, no conflicts of interest.
