# Supplementary material for: Establishing the Minimum Media Time Sample Required to Obtain Reliable Estimates of Children’s Digital Media Food Marketing Exposures
Source: Curr Dev Nutr. 2023 Apr 25;7(6):100092. doi: 10.1016/j.cdnut.2023.100092 (PMC10196768; doi:10.1016/j.cdnut.2023.100092)
Supplement: Multimedia component 1 [file mmc1.docx]

**Supplemental Table 1.** Demographic characteristics of participants in the 30%, 50% and 80% datasets

|  | **30% Dataset** | **50% Dataset** | **80% Dataset** |
| --- | --- | --- | --- |
| Participants (n) | 92 | 81 | 32 |
| **SES tertile** | | | |
| Low SES (SEIFA deciles 1-3) | 16.9% | 15.4% | 13.3% |
| Medium SES (SEIFA deciles 4-7) | 27.0% | 25.6% | 20.0% |
| High SES (SEIFA deciles 8-10) | 56.2% | 59.0% | 66.7% |
| **Gender** | | | |
| Female | 67.4% | 66.7% | 50.0% |
| Male | 32.6% | 33.3% | 50.0% |
| **Age (years)** | | | |
| 14 | 9.8% | 9.9% | 6.3% |
| 15 | 17.4% | 17.3% | 18.8% |
| 16 | 20.7% | 18.5% | 15.6% |
| 17 | 48.9% | 50.6% | 53.1% |
| 18 | 3.3% | 3.7% | 6.3% |
| **Usual weekly time on mobile devices (minutes)** | | | |
| ≤250 | 13.0% | 12.3% | 18.8% |
| 251-500 | 32.6% | 34.6% | 40.6% |
| 501-1000 | 33.7% | 35.8% | 31.3% |
| 1001-2000 | 17.4% | 13.6% | 9.4% |
| ≥2001 | 3.3% | 3.7% | 0.0% |
